# Increased HMGB1 expression and release by mononuclear cells following surgical/anesthesia trauma

**DOI:** 10.1186/cc9316

**Published:** 2010-11-02

**Authors:** Valeria Manganelli, Michele Signore, Ilaria Pacini, Roberta Misasi, Guglielmo Tellan, Tina Garofalo, Emanuela Lococo, Piero Chirletti, Maurizio Sorice, Giovanna Delogu

**Affiliations:** 1 Department of Experimental Medicine, "Sapienza" University of Rome, Viale Regina Elena 324, Rome 00161, Italy; 2 Department of Hematology, Oncology and Molecular Medicine, Istituto Superiore di Sanità, Viale Regina Elena 299, Rome 00161, Italy; 3 Department of Anesthesia and Intensive Care, "Sapienza" University of Rome, Viale del Policlinico 155, Rome 00161, Italy; 4 Laboratory of Experimental Medicine and Environmental Pathology, "Sapienza" University, Viale dell'Elettronica, Rieti 02100, Italy; 5 Department of General Surgery, "Sapienza" University of Rome, Viale del Policlinico 155, Rome 00161, Italy

## Abstract

**Introduction:**

High mobility group box 1 (HMGB1) is a key mediator of inflammation that is actively secreted by macrophages and/or passively released from damaged cells. The proinflammatory role of HMGB1 has been demonstrated in both animal models and humans, since the severity of inflammatory response is strictly related to serum HMGB1 levels in patients suffering from traumatic insult, including operative trauma. This study was undertaken to investigate HMGB1 production kinetics in patients undergoing major elective surgery and to address how circulating mononuclear cells are implicated in this setting. Moreover, we explored the possible relationship between HMGB1 and the proinflammatory cytokine interleukin-6 (IL-6).

**Methods:**

Forty-seven subjects, American Society of Anesthesiologists physical status I and II, scheduled for major abdominal procedures, were enrolled. After intravenous medication with midazolam (0.025 mg/Kg), all patients received a standard general anesthesia protocol, by thiopentone sodium (5 mg/Kg) and fentanyl (1.4 μg/Kg), plus injected Vecuronium (0.08 mg/Kg). Venous peripheral blood was drawn from patients at three different times, t_0_: before surgery, t_1_: immediately after surgical procedure; t_2_: at 24 hours following intervention. Monocytes were purified by incubation with anti-CD14-coated microbeads, followed by sorting with a magnetic device. Cellular localization of HMGB1 was investigated by flow cytometry assay; HMGB1 release in the serum by Western blot. Serum samples were tested for IL-6 levels by ELISA. A one-way repeated-measures analysis ANOVA was performed to assess differences in HMGB1 concentration over time, in monocytes and serum.

**Results:**

We show that: a) cellular expression of HMGB1 in monocytes at t_1 _was significantly higher as compared to t_0_; b) at t_2_, a significant increase of HMGB1 levels was found in the sera of patients. Such an increase was concomitant to a significant down-regulation of cellular HMGB1, suggesting that the release of HMGB1 might partially derive from mononuclear cells; c) treatment of monocytes with HMGB1 induced *in vitro *the release of IL-6; d) at t_2_, high amounts of circulating IL-6 were detected as compared to t_0_.

**Conclusions:**

This study demonstrates for the first time that surgical/anesthesia trauma is able to induce an early intracellular upregulation of HMGB1 in monocytes of surgical patients, suggesting that HMGB1 derives, at least partially, from monocytes.

## Introduction

Up to the present the stress response to an injury such as surgical/anesthesia trauma has represented a complex, poorly understood phenomenon. Nevertheless, there is a growing body of research on this important aspect of the field. Surgical/anesthesia trauma-induced stress response is mediated by a massive neuro-endocrine-hormonal flux, resulting in activation of intracellular signaling pathways and production of several molecules among which cytokines play a crucial role in regulating the function of activated cells and in preserving body homeostasis [1,2]. The intensity of such an inflammatory response is dependent on many factors, including the magnitude of tissue damage, the patient's pre-existing diseases, the type of surgery and surgeon's experience, as well as the anesthesia regimen [3,4].

In particular, anesthetic agents are suspected of impairing the perioperative inflammatory process by affecting the host cell-mediated immune balance both directly and indirectly [5]. For example, several *in vitro *and *in vivo *investigations demonstrated the direct immunosuppressive effect of volatile and non-volatile anesthetics on various lymphocyte cell lines. Moreover, drugs employed for inducing and maintaining general anesthesia, such as opioids and muscle relaxants, as well as sevoflurane, exhibited a pro-apoptotic effect on lymphocyte cells by decreasing mitochondrial transmembrane potential or activating extrinsic cell death pathways [5,6].

Recently, an endocrine family of biomolecules, termed "alarmins" by J. Oppenhaim and co-workers, is receiving growing attention as innate danger signals. High Mobility Group Box 1 (HMGB1) is a 30 KDa protein that shows all the typical features of alarmins. HMGB1 plays a pivotal role in inducing and enhancing immune cell functions as well as in tissue injury and repair [7,8].

In particular, HMGB1 was first described as a DNA-binding non-histone chromosomal protein that has been implicated in diverse cellular functions, such as stabilization of nucleosomal structure and regulation of transcription factors [9,10].

Later, several research groups showed that HMGB1 exhibits an extracellular role as a cytokine, being actively secreted by peripheral blood mononuclear cells (PBMCs). In particular, recent studies have shown a delayed release of HMGB1 by activated monocytes via a non-classical vesicle-mediated secretory pathway [11]. Functionally, HMGB1 is involved in various inflammatory processes that culminate in the release of cytokines and other inflammatory mediators [12-15]. Perhaps most of these effects are initiated by the binding of HMGB1 to the receptor for advanced glycation end products (RAGE), a multi-ligand receptor of the immunoglobulin superfamily. In addition to RAGE, members of the Toll-like receptor (TLR) family, such as Toll-like receptor 2 and 4 have been demonstrated to participate in the HMGB1 signaling pathway [16-18].

It has also been demonstrated that HMGB1 is released in the serum of subjects undergoing traumatic/surgical injury [19,20]. However, neither the kinetics of this event nor how the cellular compartment is involved in this process is actually known.

Therefore, the aim of this study was to measure HMGB1 levels in circulating monocytes as well as in the serum of patients undergoing elective surgical trauma. In addition, we evaluated a possible relationship between HMGB1 and Interleukin-6 (IL-6) production, since IL-6 is a key cytokine involved in surgical stress response.

## Materials and methods

### Patients

Following approval by the Human Subjects Review Committee and the Research Ethics Board, 47 adult subjects, American Society of Anesthesiologists (ASA) physical status I and II, scheduled for major abdominal procedures, were included in a prospective study. Patients with diabetes, cardiac, pulmonary, renal, vascular, immunologic, neurodegenerative, infectious or hepatic diseases were excluded from the study.

Subjects who were taking medication known to interfere with hormonal, metabolic or immunological function as well as pregnant or breast feeding women were also excluded.

Written informed consent was obtained from eligible patients during the screening period, at which time physical examination and medical history were evaluated. Postoperative complications were recorded throughout seven post-surgery days. Fifteen control subjects matched for sex, age and weight were also enrolled. Informed consent was obtained from the control subjects as well as the patients.

### Anesthesia technique

After intravenous medication with midazolam (0.025 mg/Kg), all patients received a standard general anesthesia protocol. Anesthesia induction was performed by thiopentone sodium (5 mg/Kg) and fentanyl (1.4 μg/Kg). Vecuronium (0.08 mg/Kg) was injected to facilitate orotracheal intubation during direct laryngoscopy.

Anesthesia was maintained with 60% air in oxygen supplemented with 1 to 2.5% inspired concentration of sevoflurane, fentanyl and vecuronium administered according to clinical need. In all patients a radial artery catheter was inserted for continuous monitoring of arterial blood pressure.

In addition, standard parameters such as electrocardiogram (ECG), oxygen saturation (SaO_2_), End-Tidal carbon dioxide (ETCO_2_) and hemoglobin (HB) were measured during surgery. All patients' lungs were mechanically ventilated by means of S/5 AVANCE device (Datex-Ohmeda, Helsinki, Finland) with the goal of achieving an ETCO_2 _level of 38 to 40 mmHg. Normal saline and Ringer Lactate solutions were administered with the infusion rate being adjusted from 6 to 10 ml/Kg/h according to blood loss. Rectal temperature was maintained at 37°C by warming fluids before administration and using an upper body Bair Hugger (Arizant Healthcare Inc., Eden Prairie, MN, USA). Duration of both surgery and anesthesia was recorded. The same surgical team performed all operative procedures.

After surgery neuromuscular blockade was antagonized with 0.5 to 1.5 mg atropine and 1 to 2.5 mg intrastigmine. Post-operative pain relief was provided by intravenous morphine bolus administered (0.20 mg/Kg) 30 minutes before the anticipated end of surgery and continued by means of elastomeric pump containing morphine 0.3 mg/Kg throughout 24 postoperative hours.

### Samples

Venous peripheral blood was drawn from patients at three different times, that is, t_0_: before anesthesia and surgery, t_1_: immediately after surgical procedure; and t_2_: at 24 hours following intervention. After allowing the blood to coagulate, the serum was isolated by low-speed centrifugation at 4°C, frozen and stored at -80°C until used. In parallel, human peripheral blood mononuclear cells were isolated from fresh heparinized blood by Lymphoprep (Nycomed AS Pharma Diagnostic Division, Oslo, Norway) density-gradient centrifugation and washed three times in phosphate buffered saline (PBS), pH 7.4.

### Isolation of monocytes

Human peripheral blood mononuclear cells were washed three times in PBS, pH 7.4. CD14+ monocytes were purified by incubation with anti-CD14-coated microbeads (Miltenyi Biotec, Bergisch Gladbach, Germany), followed by sorting with a magnetic device (MiniMacs Separation Unit; Miltenyi Biotec), according to the manufacturer's instructions [21].

### Flow cytometric analysis of HMGB1 expression

Cellular localization of HMGB1 was investigated by indirect immunofluorescence assay. Monocytes cells were collected, washed in PBS and then fixed with 2% paraformaldehyde (PFA) for 20 minutes at room temperature. The cellular suspension was then washed with cold PBS and permeabilized with 60 μM digitonin (Calbiochem, San Diego, CA, USA) in the presence of polyclonal rabbit anti-human HMGB1 (1 μg/ml, Abcam) for one hour at room temperature. After washing with cold PBS, cells were incubated with Fluorescein isothiocyanate (FITC)-conjugated goat anti-rabbit IgG (Sigma Chem Co, St Louis, MO, USA) in the presence of 60 μM digitonin for 30 minutes at room temperature.

The unbound Ab was removed by the addition of PBS containing 0.1% bovine serum albumin (BSA) and centrifugation at 5,000 g for three minutes (twice). Nonspecific binding was determined by an unlabeled isotypic control antibody (Coulter-Immunotech, Hamburg, Germany).

Cells were analyzed by flow cytometry by an Epics XL-MCL Cytometer (Coulter Electronics, Hialeah, FL, USA) equipped with a 488 nm argon-ion laser. For each histogram, 10,000 cells were counted. Antibody reactivity was reported as mean fluorescence intensity. The purity of the monocyte population was checked by staining with FITC-conjugated monoclonal antibody (MoAb) anti-CD14 (Sigma Chem Co).

Blood samples collected from 15 healthy volunteers were analysed as controls.

### Preparation of cytosolic and nuclear extracts

Monocyte cells were resuspended in buffer A (20 mM HEPES, pH 7.9, 20 mM KCl, 3.0 mM MgCl_2_, 0.3 mM Na_3_VO_4_, and freshly added 200 μM leupeptin, 10 mM E64, 300 μM PMSF, 0.5 μg/ml pepstatin, 5 mM DTT, 0.1% Nonidet P-40) and vortexed. After 30 minutes on ice, cells were centrifuged for 30 minutes at 10.000 × *g *at 4°C. The pellet was resuspended in buffer A + 0.1% Nonidet P-40 and vortexed. After centrifugation at 10,000 g for five minutes at 4°C, supernatants were taken as cytosolic extracts and frozen.

Pellets were resuspended in buffer B (40 mM HEPES, pH 7.9, 0.84 M NaCl, 0.4 mM EDTA, 50% glycerol, 0.3 mM Na_3_VO_4_, and freshly added 200 μM leupeptin, 10 μM E64, 300 μM PMSF, 0.5 μg/ml pepstatin, 5 mM DTT), and vortexed. After one hour on ice, nuclear extracts were cleared at 10,000 × *g *for one hour at 4°C and supernatants were transferred to new vials. Protein content was determined by Bradford assay using BSA as a standard (Bio-Rad Lab., Richmond, CA, USA) and samples were frozen at -80°C.

Equal amounts of nuclear or cytosolic extracts were separated in 15% SDS-PAGE under unreducing conditions. The proteins were electrophoretically transferred onto nitrocellulose membrane (Bio-Rad Lab.) and then, after blocking with PBS, containing 1% albumin, probed with monoclonal anti-HMGB1. Bound antibody visualized with HRP-conjugated anti-mouse IgG (Sigma Chem Co) and immunoreactivity was assessed by the chemiluminescence reaction, using the ECL Western blotting system (Amersham Pharmacia Biotech, Buckinghamshire, UK). As a control for purity mouse anti-α-tubulin monoclonal antibodies (Sigma Chem Co) and goat anti-laminin B polyclonal antibodies (Santa Cruz Biotechnology, Santa Cruz, CA, USA) were used.

### Immunoblotting analysis

Total protein concentration of serum and plasma samples was evaluated using the Bradford assay. Equal amounts of diluted serum samples were then subjected to sodium-dodecyl sulphate polyacrilamide gel electrophoresis (SDS-PAGE). The proteins were electrophoretically transferred onto polyvinilidene difluoride (PVDF) membranes (Bio-Rad, Hercules, CA, USA). Membranes were subsequently blocked with 5% defatted dried milk in Tris buffered saline (TBS) containing 0.05% Tween-20 and probed with anti-HMGB1 monoclonal antibody (Abcam, Cambridge, MA, USA). Bound antibodies were visualized with HRP-conjugated anti-mouse IgG (Sigma Chem Co) and immunoreactivity assessed by chemiluminescence reaction using the ECL Western blocking detection system (Amersham). Densitometric scanning analysis was performed on Mac OS 9.0 version, using NIH Image 1.62 software, developed at the U.S. National Institutes of Health [22].

We measured HMGB1 in both serum and plasma and the results were virtually the same in all the samples under test (data not shown).

### IL-6 assay

Serum samples were tested for IL-6 levels by enzyme-linked immunosorbent assay (ELISA), using a commercially available ELISA kit (R&D Systems, Inc., Minneapolis, MN, USA), according to the manufacturer's instruction. Preliminary experiments were designed to determine the detection limits as well as the linearity and range of the ELISAs, essentially in accordance with the International Conference on Harmonisation Q2A and Q2B guidelines (Committee for Proprietary Medicinal Products, European Medicines Evaluation Agency). The intra-assay variation ranged from 3% to 6% and the inter-assay variation from 4% to 9%. The limits of detection were 0.7 pg/ml. In parallel experiments, monocytes, isolated as above from the patients under test, were incubated in the presence or in the absence of 100 ng/ml recombinant histidine-tagged HMGB1 (Sigma Chem Co), 100 ng/ml lipopolysaccharide (LPS) (Sigma Chem Co) or 100 ng/ml LPS plus 100 ng/ml HMGB1 for 24 h at 37°C. IL-6 levels in the supernatant were detected by ELISA as reported above.

### Statistical analysis

Summary statistics are presented as mean and Standard deviation (SD). A one-way repeated-measures analysis ANOVA was performed to assess differences in HMGB1 concentration over time, in both monocytes and serum.

Bonferroni post tests were used to determine the significant differences between group means in an ANOVA setting. Differences were considered statistically significant when *p *was less than 0.05.

## Results

### Patients

Characteristics of patient group as well as type of surgical procedures are given in Table 1. Anesthesia/operation time and the average dosage of anesthesia drugs are reported in Table 2. None of the patients received blood transfusions during the study time as the components of transfused blood may have immunomodulatory effects in the recipient with the potential to increase or suppress production of HMGB1. Patients did not exhibit any serious post-operative complications throughout the overall study period.

**Table 1:** Patient population profile and operative procedures

No. Patients	47
Male	26
Female	21
* Age, yr	64 ± 12
* Weight (Kg)	71 ± 17
ASA (I/II)	15/32
**Surgical procedures**	
Emicolectomy	18
Isterectomy	13
Gastrectomy	9
Hepatic resection	7

*Data are expressed as mean ± standard deviation.

**Table 2:** Surgery/Anesthesia duration and total anesthesia drug doses

Surgery/Anesthesia duration (minutes)	174 ± 23/186 ± 17
**Anesthesia drugs**	
Tiopenthal (mg)	359 ± 18
Fentanyl (mg)	0.3 ± 0.09
Vecuronium (mg)	11 ± 4

*Data are expressed as mean ± standard deviation.

### Cellular HMGB1 expression

**Figure 1:**
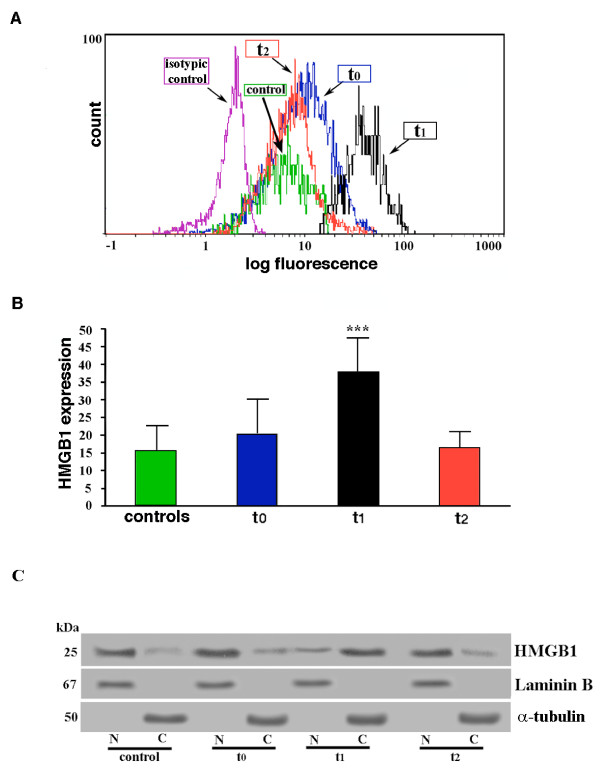
**Analysis of HMGB1 cellular expression**. **(a) **Flow cytometric analysis of HMGB1 expression in monocytes from one patient and one control subject (healthy donor). Mononuclear cells were drawn from the patients at three different times, that is, t_0_: before surgery, t_1_: immediately after surgical procedure; t_2_: at 24 hours following intervention. Cells were stained with polyclonal anti-human HMGB1 1 μg/ml (Abcam) for one hour at room temperature. Nonspecific binding was determined by an unlabeled isotypic control antibody (Coulter-Immunotech, Hamburg, Germany). After washing with cold PBS, cells were incubated with FITC-conjugated anti-rabbit IgG and then analyzed by flow cytometry. Antibody reactivity was reported as mean fluorescence intensity. Histograms show the log fluorescence versus the cell number. **(b) **Results of flow cytometric analysis of HMGB1 expression in monocytes from controls (healthy donors) and from the patients under test at three different times: t_0 _= before surgery, t_1 _= immediately after surgical procedure; t_2 _= at 24 hours following intervention. Mean fluorescence intensities were measured and plotted values represent mean ± SD. ***t_1 _vs t_0_, t_1 _vs t_2_: *P *< 0.0001. **(c) **Monocytes cells were sampled at the indicated time points and subjected to nuclear (N) and cytoplasmic (C) fractionation. The levels of endogenous HMGB1 in the nuclear and cytoplasmic fractions were determined by immunoblotting with anti-HMGB1 antibodies. Laminin B served as nuclear contamination marker and α-tubulin as cytoplasmic contamination marker. Protein loading within each compartment was also normalized with Laminin B and α-tubulin, respectively.

We first analysed HMGB1 expression level in monocytes by flow cytometry. Monocyte population was identified and gated by CD14 staining. The patients showed higher basal levels of HMGB1 than healthy donors (Figure 1a, b), consequent to the underlying diseases of the patients, but this difference was not statistically significant (*P *> 0.05). Time-course analysis revealed an increase in the mean fluorescence intensity of HMGB1 in monocytes of the patients at t_1 _(Figure 1a). Statistical analysis with all the subjects under test shows that HMGB1 staining at t_1 _is significantly higher as compared to t_0 _(*P *< 0.0001) or t_2 _(*P *< 0.0001) (Figure 1b). This finding demonstrates that HMGB1 overexpression in monocytes is an early event in surgical/anesthesia trauma.

To verify whether the enhanced expression of HMGB1 observed in monocytes may be derived by the nucleus, both cytosolic and nuclear extracts from monocytes of all the patients were probed with anti-HMGB1 Ab by Western blot. The results showed that an increased expression of HMGB1 in the cytoplasm was observed at T1 (Figure 1c). Since it was accompanied by a corresponding decrease of HMGB1 expression in the nucleus, our results suggest that neo-expression of HMGB1 in the cytoplasm may result from a translocation from the nucleus.

### Serum HMGB1 concentration

**Figure 2:**
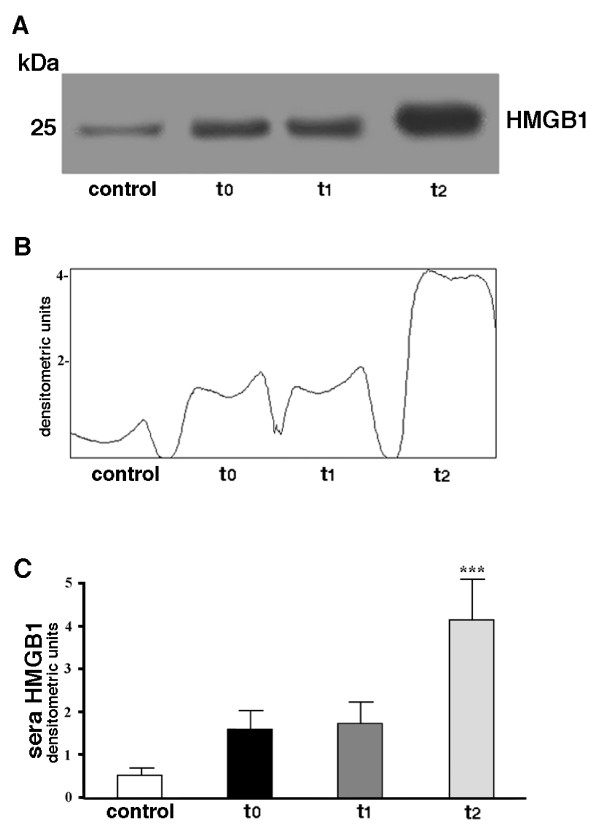
**HMGB1 serum concentration**. **(a) **Western blot analysis of serum HMGB1 concentration. Serum samples, obtained from the patients at three different times: t_0 _= before surgery, t_1 _= immediately after surgical procedure; t_2 _= at 24 hours following intervention, were analyzed by Western blot for reactivity with anti-human HMGB1 MoAb (1 μg/ml). A representative patient is shown together with a control serum from a healthy donor. **(b) **Densitometric analysis of serum HMGB1 concentration was revealed by Western blot at three different times: t_0 _= before surgery, t_1 _= immediately after surgical procedure; t_2 _= at 24 hours following intervention (arbitrary units). **(c) **Values of densitometric analyses of all the patients under test are shown as mean ± SD (arbitrary units). ***t_2 _vs t_0_, t_2 _vs t_1_: *P *< 0.001. t_1 _vs t_0_: NSS.

In parallel analyses, we detected HMGB1 levels in sera of patients at the same time points, using Western Blot (Figure 2a). Densitometric analysis (Figure 2b) revealed that HMGB1 concentration significantly increases at 24 hours (t_2_) (*P *< 0.001) (Figure 2c). On the other hand, levels of HMGB1 in serum were not significantly affected immediately after surgical procedure (t_1_) (*P *> 0.5), as compared with samples collected before surgery (t_0_). These findings provide direct evidence that overexpression of HMGB1 by monocytes precedes the increase of serum HMGB1 concentration in patients.

### Serum IL-6 concentration

**Figure 3:**
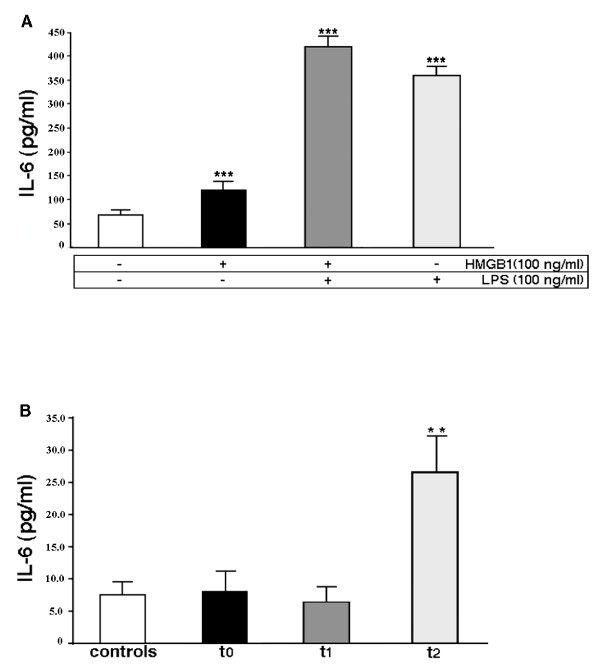
**Analysis of IL-6 levels**. **(a) **Analysis of IL-6 levels in the supernatants of monocytes from the patients under test. Monocytes were incubated in the presence or in the absence of 100 ng/ml HMGB1 or 100 ng/ml LPS plus 100 ng/ml HMGB1 for 24 h at 37°C. The samples were collected and analyzed using a commercially available enzyme-linked immunosorbent assay kit. Values are plotted as mean ± SD. ***HMGB1 vs control: *P *< 0.001; LPS vs control: *P *< 0.001; LPS plus HMGB1 vs control: *P *< 0.001. **(b) **Analysis of IL-6 levels in serum samples from the patients at three different times: t_0 _= before surgery, t_1 _= immediately after surgical procedure; t_2 _= at 24 hours following intervention. Sera from healthy subjects served as controls. The samples were collected and analyzed using a commercially available enzyme-linked immunosorbent assay kit. Values are plotted as mean ± SD. **t_2 _vs t_0 _: *P *= 0.006, t_2 _vs t_1_: *P *= 0.003. t_1 _vs t_0_: NSS.

IL-6 is commonly produced at local tissue sites and then released into circulation. Perturbation of tissue homeostasis causes IL-6 release in almost all situations and such a key cytokine is involved in surgical stress response as well. We, therefore, preliminary analyzed whether treatment of monocytes with HMGB1 induced *in vitro *release of IL-6. Monocytes from the patients under test were incubated in the presence or in the absence of HMGB1, LPS or LPS plus HMGB1. The analysis revealed that all the treatments induced a significant increase of IL-6 (*P *< 0.001) (Figure 3a), demonstrating that HMGB1 is able to trigger *in vitro *release of IL-6 by monocytes. As expected, the levels of IL-6 following LPS treatment were lower as compared to those following LPS plus HMGB1 treatment, supporting the view of a synergic action between LPS and HMGB1 [23].

Then, we tested IL-6 levels in serum samples by ELISA. The results show that this proinflammatory cytokine markedly increases at t_2 _if compared to t_0 _and t_1 _time points (t_2 _vs t_0_, *P *= 0.006; t_2 _vs t_1_, *P *= 0.003) (Figure 3b), indicating that IL-6 release is temporally related with the observed increase in HMGB1 concentration in the sera of patients.

## Discussion

This study was undertaken to investigate HMGB1 production kinetics in patients undergoing major elective surgery and to address how circulating mononuclear cells are implicated in this setting. Measurement of serum level of IL-6 allowed us to study the eventual relationship between HMGB1 and IL-6, a widely known marker of surgical stress being directly correlated with the severity of surgery and the extent of traumatic injury [20,24]. The results obtained in this work showed that: a) cellular expression of HMGB1 in monocytes immediately after the end of surgical procedure was significantly higher as compared to preoperative values; b) at 24 hours following surgery, a significant increase of HMGB1 levels was found in the sera of patients, (interestingly, such an increase was concomitant to a significant down-regulation of cellular HMGB1, suggesting that the release of HMGB1 might, at least partially, derive from mononuclear blood cells); and c) at the same time, high amounts of the circulating proinflammatory cytokine IL-6 were detected as compared to baseline preoperative levels.

These current data are consistent with previous observations demonstrating that HMGB1 is secreted by activated monocytes and is passively released by damaged cells following different types of injury, including surgical/anesthesia stress [19,20,25,26]. It is conceivable that an increase of HMGB1 in patient sera may also depend on passive protein release from damaged cells by surgical procedures as well as from intestinal manipulation leading to endotoxin translocation which in turn could induce HMGB1 release [27].

Furthermore, our findings support the view that increased levels of HMGB1 constitute an early phenomenon in traumatic insult, in contrast to the evidence reported for human sepsis as well as for experimental models of endotoxemia, in which HMGB1 is considered a late mediator [28-30]. In particular, the present study shows for the first time the intracellular overexpression of HMGB1 in monocytes of patients immediately after surgery. This finding suggests that surgical stimuli may rapidly activate intracellular pathways leading to secretion of HMGB1, which is subsequently spilled out into the circulatory stream. In fact, at 24 hours following surgery, we observed a down-modulation of cellular HMGB1in mononuclear blood cells and a significant increase of HMGB1 levels in serum. It is conceivable that an increase of HMGB1 in patient sera may also depend on a passive release of such a protein from damaged cells following surgical procedures [8]. Nevertheless, following surgical injury, monocytes display an abnormal intracellular expression of HMGB1 and this could represent an early event in surgical injury-induced stress response. The ultimate mechanism underlying regulation of this active HMGB1 release by surgical stimuli as well as the position that surgery *per se *or general anesthesia occupies in the phenomenon, still remains elusive. In this respect, it was found that Reactive Oxygen Species (ROS) were able to induce active HMGB1 secretion from monocytes in culture and hypoxic conditions or oxidative stress also trigger hepatocytes to produce HMGB1 through a calcium mediated cell signaling [31,32].

It is noteworthy that in previous works we demonstrated both overproduction of ROS by PBMCs in patients undergoing surgery and general anesthesia and the capacity of some anesthetic compounds to induce oxidative stress by altering the mitochondrial redox state [33,34]. Based on these findings, we hypothesize that the postoperative upregulation of HMGB1 is related to the impact of surgery and anesthesia on redox metabolism and subsequent increased ROS production.

Moreover, although it is known that apoptotic cells are not capable of HMGB1 release, since they retain such a molecule within their nuclear compartment it was recently demonstrated that macrophages engulfing apoptotic cells are induced to secrete HMGB1 [12]. Indeed, there is evidence that an accelerated rate of apoptosis in circulating lymphocytes occurred in the early postoperative period [35-37]. Thus, we can further hypothesize that the accelerated rate of apoptosis following surgery/anesthesia trauma, could be implicated in the massive HMGB1 release found in patients within 24 hours after a surgical procedure.

Together with an increase of circulating HMGB1, an additional finding of our study was the demonstration that: a) treatment of monocytes with HMGB1 induced *in vitro *release of IL-6; b) at t_2_, high amounts of circulating IL-6 were detected as compared to t_0_. This strongly suggests that HMGB1 postoperative increase might be able to induce IL-6 secretion. It has provided evidence that HMGB1 binds Toll-like receptor 4 (TLR-4) on monocytes surface, thus triggering a signal transduction cascade. TLR pathway activation involves the phosphorylation of myeloid differentiation factor 88 (MyD-88) and interleukin-1 receptor-associated kinase (IRAK), which in turn promotes activation and nuclear translocation of nuclear factor kB (NF-kB) ultimately leading to the release of cytokines, including IL-6 [17].

In line with our results, M.J. Cohen *et al. *found a positive correlation between IL-6 and HMGB1 levels in severely injured patients [25].

Further evidence of the potential induction of IL-6 secretion by HMGB1 comes from the studies demonstrating that HMGB1 significantly correlates with IL-6 in cerebrospinal fluid of humans. Moreover, it has been shown that intracerebroventricular administration of HMGB1 enhances brain IL-6 production in animal models [29,38].

## Conclusions

In conclusion, this study demonstrates for the first time that surgical/anesthesia trauma is able to induce an early intracellular upregulation of HMGB1 in monocytes of surgical patients. A statistically relevant increase in both IL-6 and HMGB1 serum levels at 24 h after surgery fosters the hypothesis that serum post-operative HMGB1 derives, at least partially, from monocytes and exhibits the potential to trigger IL-6 secretion. The clinical impact of these findings as well as the ultimate mechanism by which surgical/anesthesia stimuli modulate HMGB1 production, opens an interesting debate deserving of further studies.

## Key messages

• Surgical/anesthesia trauma can induce an early intracellular upregulation of HMGB1 in monocytes of surgical patients.

• HMGB1 is released in the serum of subjects undergoing traumatic/surgical injury 24 hours later.

• A role is suggested for released HMGB1 as a trigger for IL-6 secretion.

## Abbreviations

ASA: American Society of Anesthesiologists; BSA: bovine serum albumin; ECG: electrocardiogram; ELISA: enzyme-linked immunosorbent assay; ETCO_2_: End-Tidal carbon dioxide; FITC: fluorescein isothiocyanate; HB: haemoglobin; HMGB1: high mobility group box 1; IL-6: interleukin-6; IRAK: interleukin-1 receptor-associated kinase; LPS: lipopolysaccharide; MoAb: monoclonal antibody; MyD88: myeloid differentiation factor 88; NF-kB: nuclear factor kB; PBMCs: peripheral blood mononuclear cells; PBS: phosphate buffered saline; PFA: paraformaldehyde; PVDF: polyvinilidene difluoride; RAGE: receptor for advanced glycation end products; ROS: reactive oxygen species; SaO2: oxygen saturation; SD: standard deviation; SDS-PAGE: sodium-dodecyl sulphate polyacrilamide gel electrophoresis; TBS: Tris buffered saline; TLR: toll-like receptor.

## Competing interests

The authors declare that they have no competing interests.

## Authors' contributions

VM, M Signore, IP, RM, TG and EL performed research and analysed data. GT and PC selected the patients and performed clinical and laboratory analyses. M Sorice and GD designed the research and wrote the paper.
